# Interbreed variation in meiotic recombination rate and distribution in the domestic chicken *Gallus gallus*

**DOI:** 10.5194/aab-62-403-2019

**Published:** 2019-07-10

**Authors:** Lyubov P. Malinovskaya, Katerina V. Tishakova, Natalia A. Volkova, Anna A. Torgasheva, Yakov A. Tsepilov, Pavel M. Borodin

**Affiliations:** 1 Institute of Cytology and Genetics SB RAS, Novosibirsk, 630090, Russia; 2 Novosibirsk State University, Novosibirsk, 630090, Russia; 3 L. K. Ernst Federal Science Center for Animal Husbandry, Dubrovitsy, 142132, Russia

## Abstract

The efficiency of natural and artificial selection is critically dependent on the recombination rate. However, interbreed and individual variation in recombination rate in poultry remains unknown. Conventional methods of analysis of recombination such as genetic linkage analysis, sperm genotyping and chiasma count at lampbrush chromosomes are expensive and time-consuming. In this study, we analyzed the number and distribution of recombination nodules in spermatocytes of the roosters of six chicken breeds using immunolocalization of key proteins involved in chromosome pairing and recombination. We revealed significant effects of breed ($R^{2}=0.17$; p<0.001) and individual ($R^{2}=0.28$; p<0.001) on variation in the number of recombination nodules. Both interbreed and individual variations in recombination rate were almost entirely determined by variation in recombination density on macrochromosomes, because almost all microchromosomes in each breed had one recombination nodule. Despite interbreed differences in the density of recombination nodules, the patterns of their distribution along homologous chromosomes were similar. The breeds examined in this study showed a correspondence between the age of the breed and its recombination rate. Those with high recombination rates (Pervomai, Russian White and Brahma) are relatively young breeds created by crossing several local breeds. The breeds displaying low recombination rate are ancient local breeds: Cochin (Indo-China), Brown Leghorn (Tuscany, Italy) and Russian Crested (the European part of Russia).

## 1 Introduction

Recombination makes a substantial contribution to genetic and phenotypic variability. Therefore, the efficiency of natural and artificial selection is critically dependent on the recombination rate (Battagin et al., 2016; Gonen et al., 2017). It has been shown that populations with higher recombination rate demonstrate a stronger response to selection (Gorlov et al., 1992; Korol and Iliadi, 1994; Otto and Barton, 2001). Variation in recombination rate between species (Dumont and Payseur, 2008, 2011a; Smukowski and Noor, 2011) and individuals (Broman et al., 1998; Koehler et al., 2002) has been extensively studied in mammals but rather poorly in birds.

Data obtained to date indicate much higher and less variable recombination rate in birds compared to mammals (Semenov et al., 2018). High recombination rate is determined, at least partly, by a higher proportion (about two-thirds) of microchromosomes carrying at least one obligatory chiasma. Low interspecies variation in recombination rate is usually explained by a low variation in chromosome number. In most birds, it is almost the same (2n=78–80) (Griffin et al., 2007). However, Malinovskaya et al. (2018) demonstrated that the bird species that differ substantially in chromosome number may have the same recombination rate. On the other hand, the species with the same chromosome number show significant differences in recombination rate (Calderon and Pigozzi, 2006; Semenov et al., 2018).

Variation in recombination rate, which is not explained by variation in chromosome number, is especially interesting for animal breeding. Studies in mammals revealed substantial additive genetic components of such variation. The heritability of recombination rate was estimated as 0.30 in humans, 0.22 to 0.26 in cattle and 0.15 in sheep (Kong et al., 2004; Sandor et al., 2012; Johnston et al., 2016). Genes controlling global and local variation in recombination rate in humans and mice have been identified and mapped (Fledel-Alon et al., 2011; Dumont and Payseur, 2011b).

Recombination studies in poultry are less advanced. Female-, male- and sex-averaged genetic maps of chicken chromosomes based on the results of genetic recombination analysis of F1 hybrids between the red jungle fowl and the White Leghorn were compiled (Groenen et al., 2009). Using cytological methods, Rodionov et al. (1992) and Pigozzi (2001) obtained precise estimates of the rate and distribution of recombination events along individual chromosomes of female chickens. However, interbreed and individual variation in recombination rate and distribution in chicken remains unknown. Meanwhile, this information is important for understanding the effect of selection for productivity traits on the recombination rate. It may also predict the efficiency of selection for recombination.

Conventional methods used for analysis of recombination such as genetic linkage analysis, sperm genotyping and chiasma count on lampbrush chromosomes are expensive and time-consuming. Immunolocalization of MLH1, a mismatch repair protein of mature recombination nodules at the synaptonemal complexes (SCs), has proved to produce reliable estimates of the overall recombination frequency and of the distribution of recombination events along individual chromosomes. It was successfully used for studying recombination in many vertebrates (Anderson et al., 1999; Froenicke et al., 2002; Pigozzi, 2016; Segura et al., 2013).

In this paper, we used MLH1 immunolocalization to examine variation in meiotic recombination rate and distribution in domestic chicken. We chose six breeds for our comparative study. They differed in the traits they have been selected for. Cochin and Brahma are selected for meat, Brown Leghorn and Russian White for eggs, and Russian White and Pervomai for both traits. The breeds also differ in their breeding history. Three of them (Cochin, Brown Leghorn and Russian Crested) are ancient native breeds, while the other three (Pervomai, Russian White and Brahma) are relatively modern, created by crossing several native breeds.

We addressed several questions. What is the relative contribution of interbreed and individual variations in the overall variability of recombination rate? Do the interbreed differences in recombination rate depend on the particular trait or do they depend on how long each of the particular breeds has been in existence? Does the distribution of recombination events along each macrochromosome follow a stable pattern or does it vary from one breed to the next? Answering these questions might shed light on the relationships between recombination rate and selection for economical traits in poultry.

## 2 Material and methods

### 2.1 Animals

Sixteen adult five-month-old roosters of six different breeds were used in this study (Table 1). The roosters were raised and maintained at the poultry farm of the Federal Scientific Centre for Animal Husbandry under conventional conditions. Maintenance, handling and euthanasia of animals were carried out in accordance with the approved national guidelines for the care and use of laboratory animals. All experiments were approved by the Ethics Committee on Animal Care and Use at the Institute of Cytology and Genetics of the Siberian Department of the Russian Academy of Sciences (approval no. 35 of 26 October 2016).

**Table 1:** Mean (± SD) length of synaptonemal complex and MLH1 focus number in the roosters of six breeds.

Selected trait	Breed	N	N	SC length	MLH1	Genetic	Recombination
		individuals	cells	(µm)	foci	map	density
					number	length	(cM/µm SC)
						(cM)	
Meat	Cochin	3	121	250.6±67.1	60.4±4.7	3020	12.1
Meat	Brahma	3	150	228.5±58.7	62.7±5.3	3134	13.7
Eggs	Brown Leghorn	2	100	245.8±39.4	60.3±3.9	3013	12.3
Eggs	Russian White	3	153	229.5±35.7	62.5±6.1	3126	13.6
Eggs and meat	Pervomai	2	60	210.1±18.5	66.1±6.0	3303	15.7
Eggs and meat	Russian Crested	3	136	221.5±34.0	58.3±4.5	2914	13.2

### 2.2 Synaptonemal complex spreading and immunostaining

Chromosome spreads were prepared from the right testes by a drying-down method (Peters et al., 1997). Immunostaining was performed according to Anderson et al. (1999) using rabbit polyclonal anti-SYCP3 (1:500; Abcam, Cambridge, UK), mouse monoclonal anti-MLH1 (1:30; Abcam, Cambridge, UK) and human anticentromere (ACA) (1:70; Antibodies Inc., Davis, USA) primary antibodies. Primary antibody incubations were performed overnight in a humid chamber at 37 ${}^{\circ }$C. The secondary antibodies used were Cy3-conjugated goat anti-rabbit (1:500; Jackson ImmunoResearch, West Grove, USA), fluorescein isothiocyanate (FITC)-conjugated goat anti-mouse (1:30; Jackson ImmunoResearch, West Grove, USA) and aminomethylcoumarin (AMCA)-conjugated donkey anti-human (1:40; Jackson ImmunoResearch, West Grove, USA) antibodies. Antibodies were diluted in PBT (3 % bovine serum albumin and 0.05 % Tween 20 in PBS). A solution of 10 % PBT was used for blocking non-specific binding of antibodies. Secondary antibody incubations were carried out for 1 h at 37 ${}^{\circ }$C. Slides were mounted in Vectashield antifade mounting medium (Vector Laboratories, Burlingame, CA, USA) to reduce fluorescence fading. The preparations were visualized with an Axioplan 2 microscope (Carl Zeiss, Germany) equipped with a CCD camera (CV M300, JAI Corporation, Yokohama, Japan), CHROMA filter sets and ISIS4 image-processing package (MetaSystems GmbH, Altlußheim, Germany).

### 2.3 Image analysis

MLH1 signals were scored only if they were localized on synaptonemal complexes (SCs). The length of each SC and the total SC length in each spermatocyte were measured in micrometers using MicroMeasure 3.3 software (Reeves, 2001). The positions of centromeres and of MLH1 foci relative to the centromere were recorded. We identified individual SCs by their relative lengths and centromeric indices. To visualize the pattern of MLH1 foci distribution along each macrochromosome, we divided average length of the seven largest macroSCs by intervals and plotted the proportion of MLH1 foci located within each interval. To visualize the dependence of the recombination pattern on the chromosome size, we set the number of intervals for each macrochromosome proportional to the average SC length, each interval being ∼1 µm in length. We used two estimates of crossover interference: the relative distance between two neighboring MLH1 foci (the shorter the distance, the weaker the interference) and the shape parameter ν of the distribution of the distances fitted to gamma distribution. The ν values vary from 0 (no interference) to 20 (absolute interference, or only one crossover per chromosome) (Falque et al., 2009; Gauthier et al., 2011). The shape parameter ν of the distribution was estimated using CODA v.1.1 software (Gauthier et al., 2011). The STATISTICA 6.0 software package (StatSoft) was used for descriptive statistics.

The influence of interbreed and individual variations on the overall number of MLH1 foci per cell was estimated as the proportion of the variance explained by the variable ($R^{2}$) by two-way ANOVA using R software (function “aov”). Differences between the breeds in the average number of MLH1 foci per each macrochromosome and in SC length were estimated by Student's t test. p<0.05 was considered to be statistically significant. Values in the text and tables are presented as means ± SD.

## 3 Results

**Figure 1:**
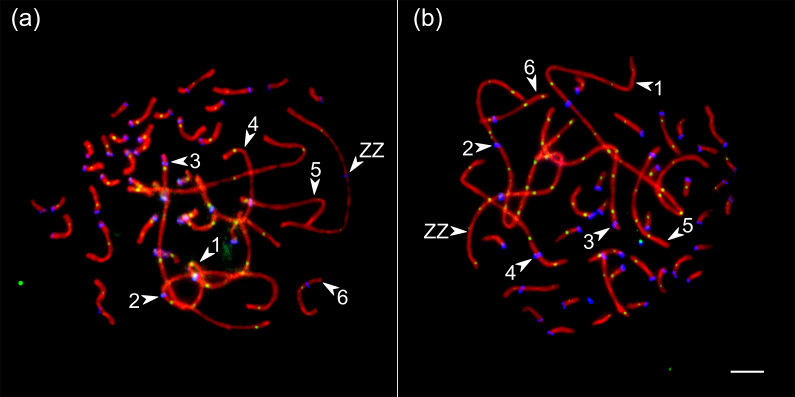
Synaptonemal complexes (SCs) of Russian Crested **(a)** and Russian White **(b)** roosters after immunolocalization of SYCP3 (red), centromeres (blue) and MLH1 (green). Arrowheads point to the SCs of the macrochromosomes identified by their lengths and centromere indices. Bar: 5 µm.

We analyzed the number and distribution of MLH1 foci at 28 080 completely synapsed SCs in 720 spermatocytes of 16 roosters. The rooster pachytene karyotype contained 38 autosomal SCs and a ZZ pair. We identified the seven largest macroSCs by their relative lengths and centromeric indices. SC1, SC2 and SCZZ were large metacentrics. They differed from each other in length and centromeric indices (p<0.001). SC3 and SC5 were large- and medium-sized acrocentics, while SC4 and SC6 were medium-sized submetacentics, which also differed from each other in their relative lengths and centromeric indices. The macroSCs 7–10 and all microSCs were acrocentric, with gradually decreasing chromosomal sizes (Fig. 1).

The breeds showed a wide variation in the total length of their SCs and the number of MLH1 per cell (Table 1). To estimate the length of the recombination map in centimorgans (cM), we multiplied the average number of MLH1 foci per cell by 50 map units (one recombination event is equivalent to 50 cM). We detected a significant effect of breed ($R^{2}=0.17$; p<0.001), but not of trait (p=0.12), on variation in MLH1 foci number. The effect of individual was also significant (ANOVA, $R^{2}=0.28$; p<0.001). Both interbreed and individual variations in MLH1 foci number were almost entirely determined by variation in MLH1 foci density on macrochromosomes, because almost each microchromosome in each breed had one MLH1 focus.

**Figure 2:**
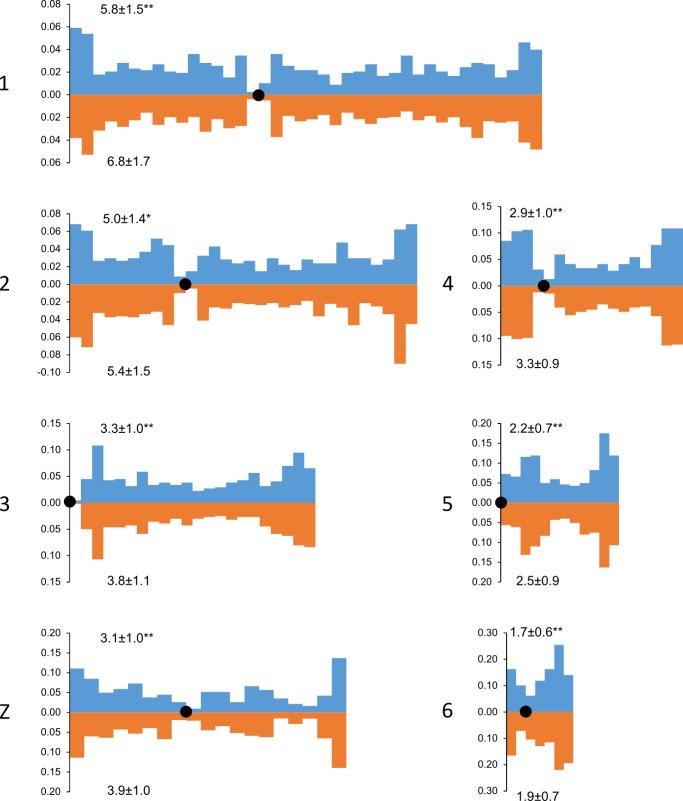
Distribution of MLH1 foci along the macrochromosomes of Russian Crested (blue columns) and Russian White (orange columns) roosters. On the x axis: positions of the MLH1 foci on the chromosomes relative to the centromere (black circle). Each interval is the proportion of the average length of each SC and is approximately 1 µm in length. On the y axis: the proportion of MLH1 foci within each interval. The numbers to the left of the y axis stand for chromosome numbers; the numbers above and below each graph show the average number of MLH1 foci at a given chromosome of a given breed. Differences between the breeds in the average number of MLH1 foci are significant (Student's t test ${}^{**}$: p<0.01; ${}^{*}$: p<0.05).

We also estimated the recombination characteristics of individual macrochromosomes: the SC length, the number of recombination nodules, the level of crossover interference (Table 2) and the pattern of MLH1 foci distribution along the SCs (Fig. 2). Table 2 shows that interbreed variation in the recombination characteristics of individual macrochromosomes was also substantial.

**Table 2:** Recombination characteristics of macrochromosomes in roosters of different breeds.

Breed	SC	cells	SC length	MLH1	Genetic	ν-	ν	Relative
		N	(µm)	foci	map	parameter	confidence	distance
				number	length		interval	between
					(cM)			MLH1 foci
Cochin	1	120	41.1±13.3	6.0±1.6	301	4.3	3.8–4.7	0.18±0.01
	2	120	34.6±11.3	5.2±1.4	261	4.4	3.9–4.9	0.21±0.01
	3	121	23.4±7.2	3.6±1.0	179	4.9	4.2–5.6	0.29±0.02
	4	121	19.2±7.5	3.2±0.9	160	4.6	3.9–5.2	0.36±0.03
	5	121	13.4±5.8	2.4±0.7	119	5.6	4.6–6.6	0.44±0.04
	6	121	7.8±3.4	1.8±0.7	90	6.4	5.0–7.8	0.52±0.03
	Z	121	21.1±7.3	3.5±0.9	176	5.2	4.5–5.9	0.31±0.03
Brahma	1	149	39.2±8.0	7.4±1.4	371	5.2	4.7–5.6	0.14±0.01
	2	149	28.8±6.0	5.5±1.1	273	5.4	4.9–6.0	0.20±0.01
	3	150	20.5±4.8	3.9±1.0	195	5.3	4.7–6.0	0.27±0.02
	4	149	16.2±3.8	3.2±0.8	158	5.1	4.4–5.7	0.36±0.03
	5	149	11.2±2.4	2.5±0.8	126	5.4	4.6–6.2	0.43±0.04
	6	145	6.5±1.5	1.9±0.5	97	6.7	5.4–7.9	0.55±0.03
	Z	149	17.9±3.4	3.8±0.9	192	5.6	4.9–6.2	0.29±0.02
Brown Leghorn	1	100	41.6±8.3	6.8±1.3	342	5.1	4.5–5.6	0.16±0.01
	2	96	31.3±6.2	5.2±1.0	260	6.3	5.5–7.1	0.21±0.01
	3	100	22.4±4.4	3.8±0.8	188	5.8	4.9–6.6	0.29±0.02
	4	99	17.5±3.7	3.1±0.7	157	6.0	5.0–7.0	0.38±0.02
	5	100	12.2±2.9	2.4±0.6	121	7.4	5.9–8.8	0.46±0.03
	6	97	7.1±1.3	1.8±0.5	89	7.5	5.7–9.4	0.55±0.02
	Z	100	21.7±4.1	3.8±0.8	190	5.0	4.3–5.7	0.30±0.02
Russian White	1	149	39.9±7.3	6.8±1.7	342	4.0	3.6–4.3	0.15±0.01
	2	149	29.9±7.0	5.4±1.5	268	4.4	4.0–4.8	0.20±0.01
	3	149	21.5±5.0	3.8±1.1	188	4.5	4.0–5.0	0.28±0.03
	4	149	16.6±4.5	3.3±0.9	163	4.8	4.2–5.4	0.35±0.03
	5	150	11.9±3.8	2.5±0.9	125	4.3	3.7–5.0	0.38±0.03
	6	147	7.3±2.6	1.9±0.7	95	5.4	4.4–6.4	0.50±0.03
	Z	149	21.8±4.3	3.9±1.0	194	4.1	3.6–4.6	0.28±0.02
Pervomai	1	57	33.3±5.2	8.2±2.0	410	4.7	4.1–5.3	0.13±0.00
	2	53	24.5±4.1	6.1±1.2	307	5.4	4.6–6.3	0.17±0.01
	3	53	18.6±3.5	4.7±1.0	235	5.6	4.6–6.7	0.22±0.01
	4	56	14.0±2.8	3.8±0.9	192	7.0	5.6–8.4	0.29±0.02
	5	47	10.3±2.1	3.0±0.8	148	5.6	4.2–7.0	0.35±0.03
	6	55	6.0±0.9	2.2±0.5	108	6.7	4.8–8.6	0.48±0.03
	Z	30	17.4±2.4	4.5±0.9	227	5.4	4.1–6.7	0.24±0.01
Russian Crested	1	134	37.0±8.4	5.8±1.5	288	3.8	3.4–4.2	0.18±0.01
	2	135	29.4±7.6	5.0±1.4	250	3.8	3.4–4.2	0.21±0.02
	3	135	20.3±4.6	3.3±1.0	164	4.4	3.8–4.9	0.31±0.03
	4	134	16.5±4.7	2.9±1.0	144	4.1	3.5–4.8	0.37±0.05
	5	133	11.5±2.9	2.2±0.7	112	5.5	4.5–6.5	0.45±0.03
	6	135	6.7±2.0	1.7±0.6	84	6.2	5.3–8.3	0.53±0.03
	Z	135	17.8±4.6	3.1±1.0	157	3.8	3.3–4.3	0.33±0.03

Despite the interbreed differences in MLH1 foci density, the patterns of their distribution along homologous chromosomes were strikingly similar (Fig. 2). We observed an increase in MLH1 foci frequency in the distal regions of all macrochromosomes. The lack of MLH1 foci in proximal regions (centromeric interference) was limited to a small pericentromeric area of about 1–2 µm in length.

Chicken macrochromosomes showed a positive correlation between SC length and number of MLH1 sites (R=0.92, p<0.001), which is typical of vertebrate chromosomes. There was a negative correlation between SC length and degree of crossover interference estimated through the relative distances between adjacent MLH1 sites (R=-0.56, p<0.05) and through the ν parameter of gamma distribution of the distances (R=-0.61, p<0.05).

## 4 Discussion

In this study, we estimated the number and distribution of recombination events in the rooster genome by immunolocalization of MLH1 protein, a reliable marker of mature recombination nodules. The main advantage of this approach over chiasma count consists in the possibility to analyze a large number of both oocytes and spermatocytes, while the general estimates of recombination rate obtained by these two methods are rather close. Rodionov et al. (1992) estimated the total number of chiasmata per chicken oocyte as 59–64, and Pigozzi (2001) estimated the number of MLH1 foci per oocyte as 65.0±4.0. In our study, the number of MLH1 foci per spermatocyte varied from 58.3±4.5 to 66.1±6.0 across the chicken breeds, with the general average being 61.5±5.6.

The results of cytological and genetic analyses of recombination are in a good agreement with each other. For example, Groenen et al. (2009) estimated the male chicken genetic map length as 3145 cM based on the results of genetic linkage analysis of F1 hybrids between the red jungle fowl and the White Leghorn. The breed-averaged male chicken genetic map resulting from our study was just 1 % shorter (3087 cM). Thus, we recommend the immunolocalization of MLH1 protein as a fast, affordable and reliable method for recombination analysis in poultry.

The most important and interesting results of our study are (1) the detection of wide individual and interbreed variation in recombination rate (both overall and macrochromosome-specific) and (2) a remarkable stability of the pattern of distribution of recombination events along macrochromosomes.

The divergence of the breeds in recombination rate may have been driven by differences in breeding histories. The breeds examined showed an interesting correspondence between the age of the breed and its recombination rate. Those with high recombination rate are relatively young breeds created by crossing several local stocks. The Pervomai breed was produced by complex reproductive crossing of three crossbred breeds: White Wyandotte (derived from crosses between Brahmas and Hamburgs), Rhode Island (derived from crosses between Malays and brown Italian Leghorns) and Yurlov Crower (derived from crosses of Chinese meat chicken, gamecocks and landraces) in 1930–1960. The Russian White originated from crosses of the White Leghorn with local chickens in 1920–1940. The Brahma breed was developed in the United States in 1840–1850 by crossing the ancient Asian breeds Cochin and Chittagong. On the other hand, the breeds displaying low recombination rate are ancient local breeds: Cochin (Indo-China), Brown Leghorn (Tuscany, Italy) and Russian Crested (the European part of Russia) (Paronyan and Yurchenko, 1989; Scrivener, 2006, 2009).

We may speculate that the correspondence between recombination rate and breed's age appears to reflect a correspondence between recombination rate and genetic heterogeneity because genetic heterogeneity within each breed tends to decrease with time due to inbreeding and artificial selection (Lipinski et al., 2008; Gibbs et al., 2009). This hypothesis is consistent with the assumption that benefits of recombination (generation of new allele combinations) prevail over its costs (occurrence of deleterious mutations) as long as the population remains sufficiently heterogeneous (Kim and Stephan, 2000; Nachman, 2001; Ohta, 1999). Thus, depletion of genetic variability may lead to a decrease in recombination efficiency, which reduces selection pressure on high recombination rate. This would in turn decrease recombination rate. Further studies are needed to check these hypotheses.

The conservatism in the distribution of recombination events across the rooster macrochromosomes confirms the pattern previously described in mammals: the recombination frequency in certain regions of vertebrate chromosomes depends more upon the position of these regions on the chromosome (that is, upon the distance from the centromere and the telomere) than upon the genetic composition of these regions (Gorlov et al., 1991; Vozdova et al., 2016; Ruiz-Herrera et al., 2017).

Our results are relevant to the current discussion about the role of recombination in selection for productivity traits. Because recombination reduces linkage disequilibrium between quantitative trait loci, it is considered a means to cope with negative epistasis and to deal with the selection plateau. It has been shown with a variety of model organisms and by computational experiments under different scenarios (Comeron et al., 1999; Pál et al., 2001; Carvalho and Clark, 2002; Simmonds, 2006; Hill and Robertson, 2008) that an increase in recombination rate enhances the efficiency of selection. However, the magnitude of this effect is still the subject of a debate. A recent simulation study by Battagin et al. (2016) demonstrated that the effect will be taking place in the distant future and requires an unrealistically high increase in global recombination rate. The simulation shows that a 2-fold increase in recombination rate leads to a 12.5 % increase in cumulative selection response over 40 generations, and a 20-fold increase in recombination rate results in a 33.4 % increase in the response to selection.

In our study, the difference in recombination rate between the most contrasted breeds, Pervomai and Russian Crested, was 19 %. Moreover, the results of our study demonstrate that the interbreed differences in recombination rate have no effect on the distribution of recombination nodules. Their localization along the chicken macrochromosomes is chromosome-specific, positionally determined and conserved between the breeds. This indicates that selection of overall recombination rate in chickens is unlikely to affect the efficiency of selection for productivity within the breeds. Apparently, the selection success could be achieved by manipulation of local rather than general recombination rate. The detection and mapping of hot and cold spots, which show up as 1000-fold differences in recombination rate, and the proteins involved in their regulation could make this approach feasible (Elferink et al., 2010; Gonen et al., 2017; Singhal et al., 2015). This targeted approach might help to break up the ancestral linkage between plus and minus alleles for productivity traits.

## Data Availability

The data sets are available upon request from the corresponding author.
